# Atypical Neuroleptic Malignant Syndrome in a COVID-19 Intensive Care Unit

**DOI:** 10.7759/cureus.27923

**Published:** 2022-08-12

**Authors:** Joshua R Durbach, Gerald Rosen, Carolina De La Cuesta, Seth Gottlieb

**Affiliations:** 1 Anesthesiology, Critical Care and Internal Medicine, Mount Sinai Medical Center, Miami, USA; 2 Anesthesiology, Mount Sinai Medical Center, Miami, USA; 3 Surgical Critical Care, Mount Sinai Medical Center, Miami, USA; 4 Pulmonary and Critical Care Medicine, Mount Sinai Medical Center, Miami, USA

**Keywords:** dantrolene, severe covid-19, difficult diagnosis, critical thinking, neuroleptic malignant syndrome (nms), second generation induced neuroleptic malignant syndrome, critical care anesthesiology, medical intensive care unit (micu), critical care and internal medicine education, sars-cov-2

## Abstract

Neuroleptic malignant syndrome (NMS) has been defined as a life-threatening neurologic emergency related to the use of antipsychotic medications. It is most often seen with high-potency (first-generation) antipsychotic medications and may occur after a single dose. There have been conflicting reports in the literature of an atypical NMS (ANMS) presentation, associated with lower-potency agents (second generation) antipsychotic medications. NMS is usually diagnosed with a tetrad of clinical symptoms although none of the tetrads is needed for diagnosis. We report a case of a patient admitted for severe acute syndrome coronavirus 2 (SARS-CoV2) pneumonia who developed probable ANMS. SARS-CoV2 also referred to as coronavirus disease 2019 (COVID-19) added another dimension of complication to patient care as we have, at this time, an incomplete understanding of the pathogenesis. We feel critical care clinicians should maintain broad differentials to clinical findings, during the use of multiple medications and not simply attribute the various presentations to COVID-19.

## Introduction

Neuroleptic malignant syndrome (NMS) has been defined as a life-threatening neurologic emergency involving multiple systems related to the use of antipsychotic medications [1]. It is most often seen with high-potency (first-generation) antipsychotic medications and may occur after a single dose [1-3]. There have been conflicting reports in the literature of an atypical NMS (ANMS) presentation, associated with lower-potency agents (second generation) antipsychotic medications [4]. We report a case of a patient admitted for severe acute syndrome coronavirus 2 (SARS-CoV2) pneumonia induced acute respiratory distress syndrome (ARDS) who developed probable ANMS.

## Case presentation

**Figure 1:**
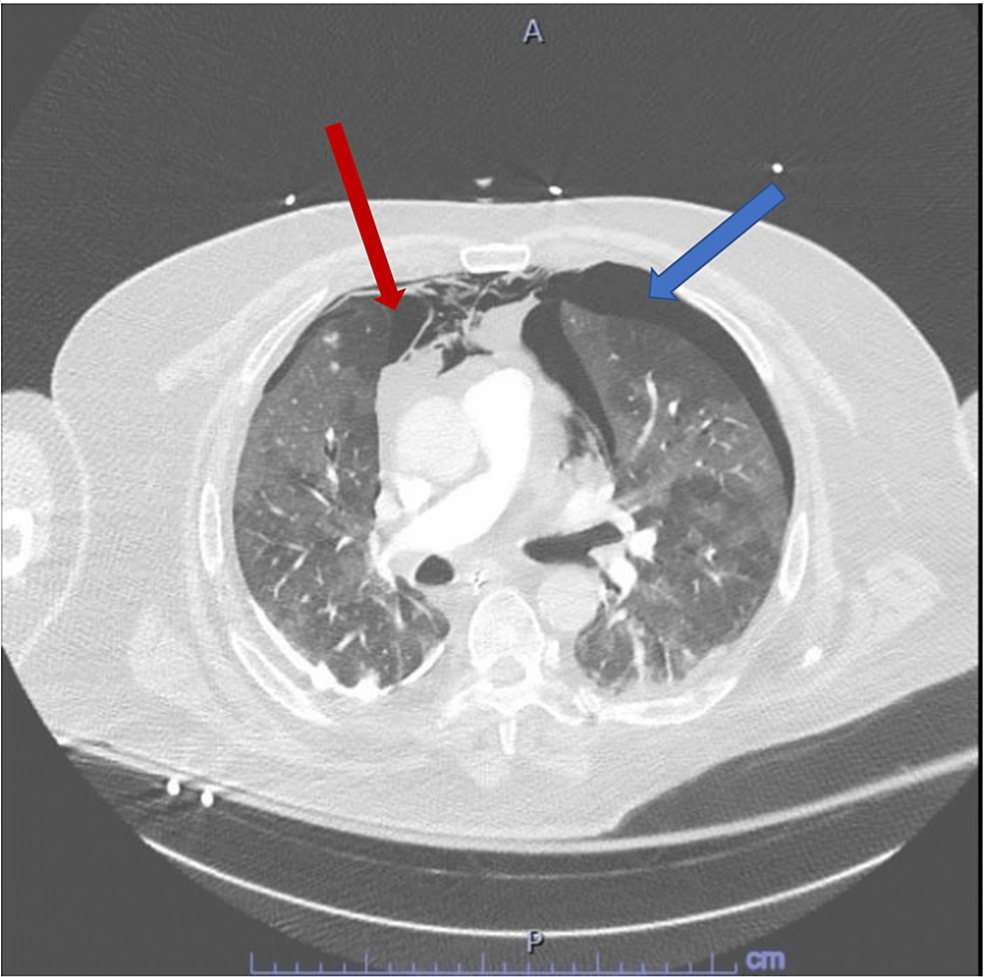
CTPA, cross-sectional view, lung window. Blue arrow left side pneumothorax, red arrow right side pneumothorax. CTPA, CT pulmonary angiogram

A 68-year-old male was admitted to the intensive care unit (ICU) due to SARS-CoV2 pneumonia, requiring a high flow nasal cannula initially. The patient’s respiratory status continued to worsen over the subsequent day leading to endotracheal intubation, mechanical ventilation, and IV sedation with midazolam and dexmedetomidine as well as a fentanyl patch every 72 h. This was titrated and done in order to meet his oxygen and ventilatory demands. Several days following the intubation, we attempted weaning trials as the patient had clinically improved. We lowered his IV sedation of midazolam, stopped his dexmedetomidine, and continued the fentanyl patch. Shortly after the aforementioned, the patient developed agitation and was started on Quetiapine 25 mg twice a day for suspected ICU delirium. The patient continued to display signs of agitation, thus he was restarted on his IV sedation as above. More than 24 h after starting Quetiapine, the patient developed new-onset tachycardia and ventilator desynchrony with worsening hypoxia, increased peak pressure, and plateau pressure along with tachypnea. The ventilator settings were adjusted to achieve the most desirable for the patient. He was given a fluid challenge which did not improve the tachycardia as an initial thought was dehydration/hypovolemia due to his increased work of breathing. Ventilator settings were changed from pressure assist control to volume assist control to meet his demands. An electrocardiogram (EKG) was obtained to characterize the tachycardia, which demonstrated sinus tachycardia with right axis deviation, compared to his previous EKGs as these were new changes. Given his new onset ventilator desynchrony, elevated peak pressure, tachycardia and tachypnea, a concern for possible pneumothorax was high on the differential along with the possibility of pulmonary embolism (PE) given the hypercoagulable status seen in SARS-CoV2. The decision to rule out both (pneumothorax and PE) with a CT pulmonary angiography was done with the CT scan leading to the diagnosis of large left and small right apical pneumothoraxes but no PE was found (Figure 1).

**Figure 2:**
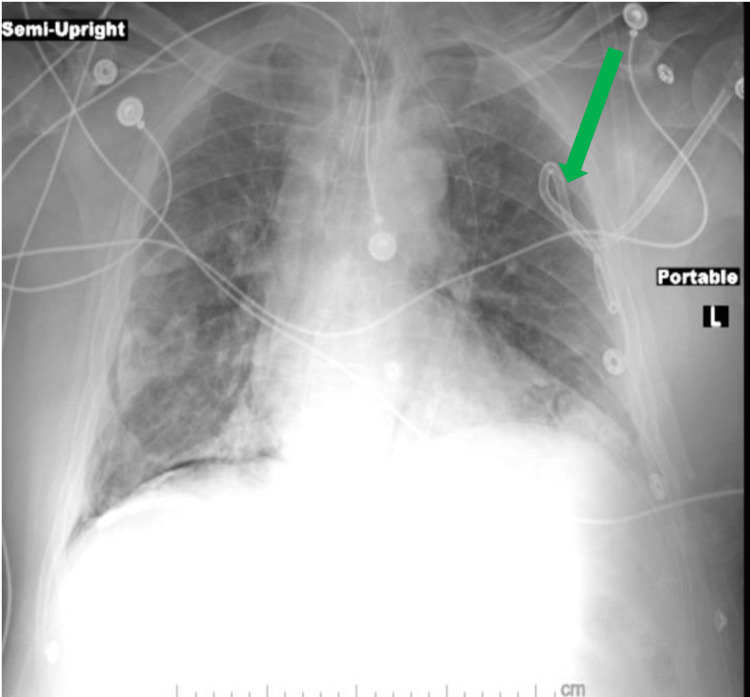
Chest radiograph, with left-sided chest tube in place and improvement of pneumothorax. Green arrow chest tube. A persistent right pneumothorax that did not require decompression.

Under sterile technique, a chest tube was inserted to decompress the left pneumothorax (Figure 2).

followed by the patient developing a persistent fever of 38.2°C. Blood (aerobic and anaerobic), urine, and tracheal aspirate cultures were ordered and obtained. The patient was previously on doxycycline 100 mg twice daily but started on broad-spectrum antimicrobial coverage with vancomycin, cefepime, and micafungin given the new febrile state. The patient’s fever continued to increase peaking at 42°C with a rectal temperature probe. He was treated with acetaminophen several times, which failed to control the pyrexia. Cooling blankets and cool saline gastric lavages were administered but these efforts failed to control his pyrexia. The patient was not on IV paralysis and rigors were not observed. Furthermore, the patient’s sinus tachycardia worsened, he became hemodynamically unstable and required IV vasopressors to maintain appropriate mean arterial pressures. A review of the patient’s recent exposures demonstrated that he was started on Quetiapine 36 h prior to these new onset features. The concern for NMS) was raised except the patient lacked the classic presentation of muscular rigidity. Therefore, we obtained, without delay, serum laboratory studies to rule out NMS along with other etiologies. The laboratory results returned with the following significant findings found in Table 1.

**Table 1:** Serum laboratory results on the day of fever and on the subsequent day laboratory results for comparison. WBC, white blood cell; RBC, red blood cell; BUN, blood urea nitrogen; AST, aspartate aminotransferase; ALT, alanine transaminase; EGFR, estimated glomerular filtration rate; TSH, thyroid stimulating hormone; CPK, creatine phosphokinase; LDH, lactate dehydrogenase

Component			Reference range and units	Value			
				Day of presenting fever		Subsequent day	
WBC count			4.80-10.80 10^3/uL	23.14		18.65	
RBC count			4.63-6.08 10^6/uL	5.43		4.7	
Hemoglobin			14.0-18.0 g/dL	16.4		14	
Hematocrit			42.0-52.0%	50.7		44.1	
Mean corpuscular volume			79.0-92.2fL	93.4		93.8	
Red cell distribution width			11.5-15%	15.3		15.3	
Platelet count			150-450 10^3/uL	174		133	
Lymphocytes			16.0-45.0%	5.8		4.2	
Neutrophils relative percent			42.0-75.0%	80.4		80.5	
Monocytes			2.0-12.0%	11.5		14.1	
Eosinophils			0.0-5.0%	0		0	
Basophils			0.0-2.0%	0.3		0.2	
Sodium			136-145 mmol/L	143		144	
Potassium			3.5-5.1 mmol/L	5.6		5.1	
Chloride			98-107 mmol/L	109		111	
Carbon dioxide			21-32 mmol/L	28		29	
Glucose			74-106 mg/dL	437		433	
BUN			7.0-18 mg/dL	76		64	
Creatinine			0.70-1.30 mg/dL	1.59		1.55	
Calcium			8.5-10.1 mg/dL	8		7.8	
AST			15-37 U/L	-		107	
ALT			16-61 U/L	-		73	
Protein, total			6.4-8.2 g/dL	-		5.9	
Albumin			3.4-5 g/dL	-		2.2	
Globulin			2.3-3.5 g/dL	-		2.7	
Alkaline phosphatase			45-117 U/L	-		94	
Bilirubin, total			0.3-1.00 mg/dL	-		1.4	
Anion gap			5.0-15.0 mmol/L	6		4	
EGFR			>60 mL/min/1.73m2	45		35	
D-dimer			0.27-0.50 ug/mL	1.1		1.2	
Ferritin			26.0-388.0 ng/mL	1323.9		2068.7	
TSH			0.358-3.740 uiu/mL	-		0.107	
Triglycerides			30-150 mg/dL	-		345	
CPK			39-308 U/L	966		2044	
LDH			84-286 U/L	1099		-	

The patient’s core body temperature remained elevated between 40°C and 42°C despite our above-mentioned treatments. Finally, a decision was made based on the patient’s presentation, pyrexia and laboratory findings, to treat for NMS. Quetiapine was discontinued and Dantrolene 1 mg/kg was administered followed by a repeat dose. Within a couple of hours of the aforementioned treatment, the patient’s vital signs normalized with resolution of his pyrexia. The patient did not develop further febrile states, autonomic instability, or tachycardia. Two days after this, one of the blood cultures grew *Streptococcus anginosus*, his antibiotics were changed to Ceftaroline by infectious disease. The patient had resolution of the pneumothoraxes but unfortunately, the patient succumbed two weeks later. 

## Discussion

The NMS is particularly rare and a disastrous drug-induced febrile state, with incident rates ranging from 0.02% to 3% among patients taking antipsychotic medications [1]. The presentation of NMS can vary without a specific diagnostic test, thus making the diagnosis challenging.

One usually suspects NMS when a patient presents with a tetrad of symptoms evolving over one-to-three days following the commencement of medication. This tetrad consists of the following clinical findings notably: 1) fever, 2) muscle rigidity, 3) encephalopathy, and 4) autonomic instability (tachycardia, labile, or high blood pressures) [2]. At this time the cause of NMS remains unknown but it is suspected to be related to dopamine receptor blockade given the correlation with antipsychotic medications, yet this does not explain the NMS presentation entirely. Adnet et al. have proposed a theory that the rigidity and tremors may be a result of nigrostriatal dopamine pathway activation, but similar to Leveson, this theory of the etiology does not explain NMS presentation wholly [2-4]. Besides the aforementioned possible causes, there may be a genetic predisposition, given the report of families experiencing NMS [5]. The genetic predisposition theory was supported in a 2003 publication by Mihara et al. who found a higher dopamine-2 receptor gene allele in patients with NMS which results in reduced dopaminergic activity and metabolism [6]. 

There are no specific laboratory blood tests one can use to rule out NMS, making the diagnosis very challenging; however, clinicians may use various laboratory blood tests to support the diagnosis by ruling out other etiologies. Certain notable laboratory blood tests one may encounter are elevated creatine kinase, leukocytosis, elevated lactate dehydrogenase, transaminases, electrolyte disturbances, rhabdomyolysis, and low serum iron concentration; as demonstrated in our patient but all non-specific and have a plethora of other etiologies for elevations. With regard to our case, it was complicated by the patient already being intubated and sedated making it difficult to determine encephalopathy (a component of the clinical tetrad), the patient was being treated with corticosteroids for COVID-19, producing a leukocytosis and the patient was already being sedated with a midazolam drip which could blunt the typical rigidity one encounters with NMS, as benzodiazepines have been used as adjunctive therapy in NMS. With this in mind, our impression was an atypical presentation of NMS, and cases of atypical NMS (ANMS) lack, most notably rigidity; usually the key clinical sign pointing you to the diagnosis. NMS and/or ANMS is usually a diagnosis of exclusion as in this case no identifiable cause was found supporting ANMS. Although one of the blood cultures later returned positive, it is unlikely that the bacteremia would cause the persistent degree of fever the patient experienced furthermore; the patient was being treated with fentanyl patches and midazolam, making withdrawal less likely. Still, our team of physicians was hesitant to accept this diagnosis due to the absence of rigidity. However, the patient only responded (resolution of the pyrexia and hemodynamic instability) once Dantrolene was administered; supporting the theory of ANMS. One must add, careful consideration was taken to administer Dantrolene as it is not without side effects. A review article in Anesthesia, states the most commonly described side effects are muscle weakness in 22% of patients, phlebitis in 10% of patients, and respiratory failure in 3% of the patients [7]. These side effects did not outweigh the benefits at the time of the patient’s presentation and thus administered. In Picard et al.’s 2008 publication, they note that NMS is typically characterized by muscle rigidity, pyrexia, autonomic dysfunction and encephalopathy, however, none of these is pathognomic, and not all four of the aforementioned are required for diagnosis [8]. One can suspect that atypical NMS has an even higher morbidity and mortality compared to typical NMS and this can be attributed to a delay in diagnosis, especially when there is a concomitant COVID-19 infection. In this case, we promote vigilance to maintain broad differentials with critically ill patients in the ICU. We remind practitioners of possible adverse reactions with the multiple medications we are using and interactions with the variety of COVID-19 medication cocktails being implemented. 

## Conclusions

Neuroleptic malignant syndrome is particularly rare and a disastrous antipsychotic drug induced febrile state that requires early recognition and diagnosis. NMS is a clinically diagnosed condition although none of the tetrad is needed for diagnosis and there are no sensitive and/or specific laboratory tests to rule out or in NMS. Based on the lack of rigidity and inability to assess mental status, this case can be considered an atypical presentation otherwise referred to as ANMS given that the patient responded briskly to Dantrolene. Moreover, COVID-19 added another dimension of complication to patient care as we have, at this time, an incomplete understanding of the pathogenesis. We promoted that critical care clinicians should maintain broad differentials to clinical findings and not simply attribute the various presentations simply to COVID-19 while managing patient in the ICU with multiple medications.

## References

[1] Velamoor R (2017). Neuroleptic malignant syndrome: a neuro-psychiatric emergency: Recognition, prevention, and management. Asian J Psychiatr.

[2] Levenson JL (1985). Neuroleptic malignant syndrome. Am J Psychiatry.

[3] Henderson VW, Wooten GF (1981). Neuroleptic malignant syndrome: a pathogenetic role for dopamine receptor blockade?. Neurology.

[4] Adnet P, Lestavel P, Krivosic-Horber R (2000). Neuroleptic malignant syndrome. Br J Anaesth.

[5] Otani K, Horiuchi M, Kondo T, Kaneko S, Fukushima Y (1991). Is the predisposition to neuroleptic malignant syndrome genetically transmitted?. Br J Psychiatry.

[6] Mihara K, Kondo T, Suzuki A (2003). Relationship between functional dopamine D2 and D3 receptors gene polymorphisms and neuroleptic malignant syndrome. Am J Med Genet B Neuropsychiatr Genet.

[7] Krause T, Gerbershagen MU, Fiege M, Weisshorn R, Wappler F (2004). Dantrolene--a review of its pharmacology, therapeutic use and new developments. Anaesthesia.

[8] Picard LS, Lindsay S, Strawn JR, Kaneria RM, Patel NC, Keck PE Jr (2008). Atypical neuroleptic malignant syndrome: diagnostic controversies and considerations. Pharmacotherapy.

